# Insights Into Mammary and Extramammary Paget’s Disease: Diagnosis, Management, and Recent Advances

**DOI:** 10.7759/cureus.80531

**Published:** 2025-03-13

**Authors:** Camila Ortuz Lessa, Fernando Fernández Varela Gómez, Víctor Hugo Garzón Ortega, Alicia Sandoval García, Katherine López Soto, Nicolás Ricardo Brito Brito

**Affiliations:** 1 Department of Dermatology, Hospital General de México Dr. Eduardo Liceaga, Mexico City, MEX; 2 Department of Surgery, Faculty of Medicine, Universidad Nacional Autónoma de México, Mexico City, MEX; 3 Department of General Surgery, Christus Muguerza Hospital Alta Especialidad, Monterrey, MEX; 4 Department of Physiology, Faculty of Medicine, Universidad Nacional Autónoma de México, Mexico City, MEX; 5 Department of Internal Medicine, Hospital Central Sur de Alta Especialidad, Mexico City, MEX; 6 Department of Plastic Surgery, Hospital General Dr. Manuel Gea González, Mexico City, MEX

**Keywords:** extramammary paget’s disease, her2 overexpression, mammary paget's disease, multidisciplinary approach, surgical excision of tumour, targeted therapy

## Abstract

Mammary Paget’s disease (MPD) and extramammary Paget’s disease (EMPD) are rare cutaneous disorders associated with adenocarcinoma, each presenting unique clinical challenges. MPD typically indicates an underlying breast cancer, manifesting as eczematous changes in the nipple-areolar complex. In contrast, EMPD appears as erythematous, scaly plaques in apocrine-gland-rich areas such as the vulva, perianal region, or axilla, with inconsistent links to internal malignancies. These conditions often resemble benign skin diseases, complicating timely diagnosis. Diagnosis involves clinical evaluation, imaging, and histopathological confirmation of Paget cells. Pathogenesis may stem from in situ malignant transformation or migration of cancer cells from distant sites, influenced by molecular pathways like HER2 and PI3K-AKT-mTOR. Treatment varies: MPD often requires mastectomy and systemic therapies, while EMPD is managed with surgical excision (e.g., Mohs surgery), adjusted to disease extent. The prognosis hinges on stage, molecular features, and associated tumors, particularly impacting MPD survival. Recent advances in precision medicine and immunotherapy, especially for EMPD, offer promising directions. This review highlights the distinct features, diagnostic hurdles, treatment strategies, and the essential role of a multidisciplinary approach in improving outcomes.

## Introduction and background

**Figure 1:**
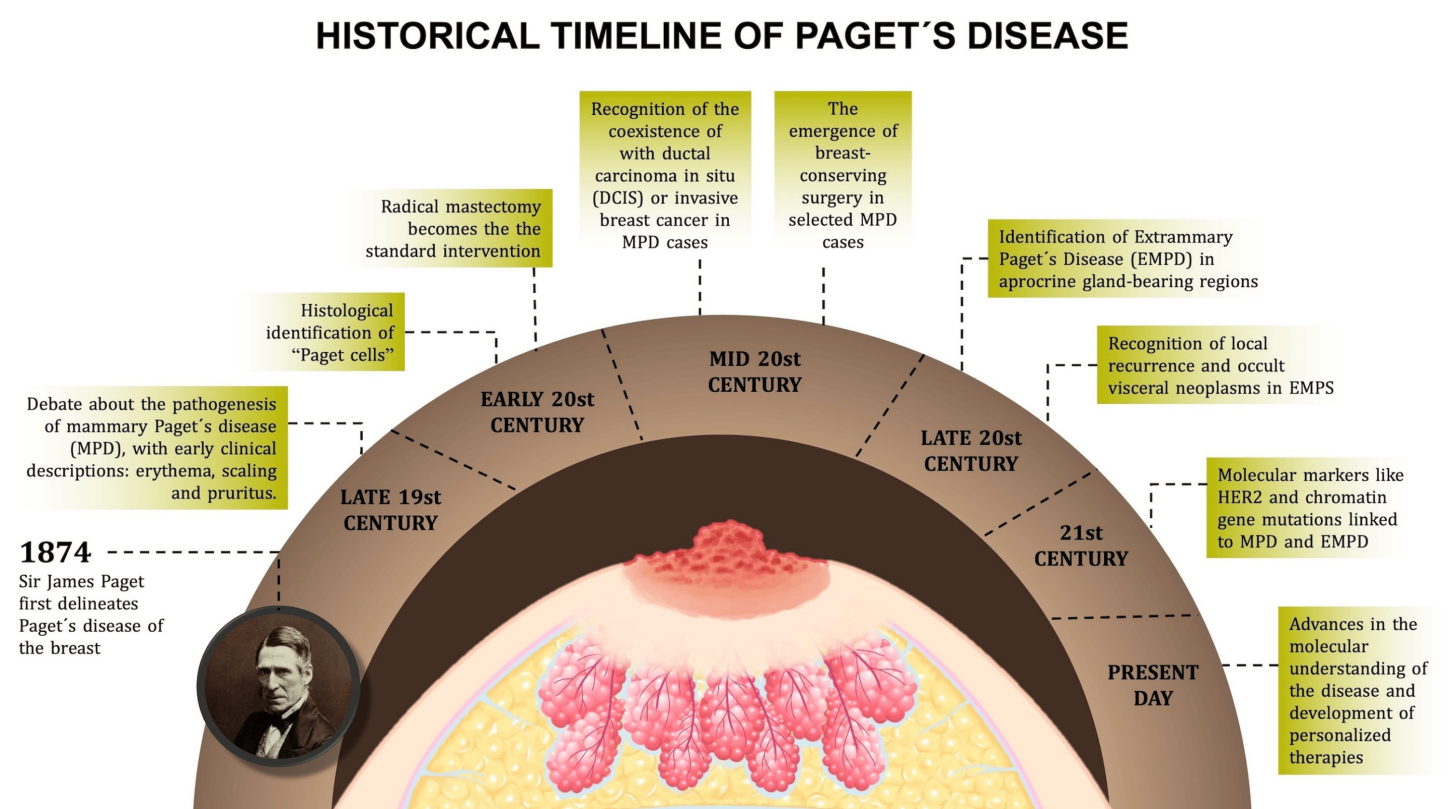
Historical Timeline of Paget’s Disease This figure presents a historical timeline of Paget’s disease of the breast, tracing key milestones from its discovery to modern advancements. The timeline begins in 1874 with Sir James Paget’s initial clinical description. It progresses through the late 19th century, noting debates on the pathogenesis of MPD and early symptom descriptions (e.g., erythema, scaling, pruritus). The early 20th century marks the histological identification of Paget cells, while the mid-20th century highlights the association of MPD with DCIS or invasive breast cancer. The late 20th century introduces breast-conserving surgery for select MPD cases and the recognition of EMPD in apocrine gland-bearing regions, alongside risks of local recurrence and occult visceral neoplasms in EMPD. The 21st century features molecular discoveries, such as Human Epidermal Growth Factor Receptor 2 (HER2) overexpression and chromatin remodeling gene mutations, linked to MPD and EMPD, culminating in present-day personalized therapies and enhanced molecular understanding. Image credit: Katherine López Soto.

Mammary Paget’s disease (MPD) and extramammary Paget’s disease (EMPD) are rare dermatological manifestations of adenocarcinoma that have intrigued clinicians and researchers since their initial descriptions. MPD was first characterized by Sir James Paget in 1874 as a condition marked by eczematous changes in the nipple-areolar complex, often signaling an underlying ductal carcinoma in situ (DCIS) or invasive ductal carcinoma (IDC) [1]. This seminal observation laid the groundwork for recognizing MPD as an intraepidermal extension of malignant glandular epithelial cells known as Paget cells. In contrast, EMPD emerged later in medical literature, identified in apocrine-rich regions such as the perianal area, genitalia, and axillae, with its recognition evolving through the early 20th century [2]. While EMPD shares histopathological similarities with MPD, its association with underlying malignancies is less consistent, ranging from primary cutaneous origins to secondary manifestations of internal cancers (Figure 1) [3].

Over time, our understanding of these conditions has progressed from clinical observations to a sophisticated grasp of their molecular and genetic foundations, including mutations in chromatin remodeling genes and the involvement of the Musashi 1-mammalian target of rapamycin (Msi1-mTOR) pathway [4,5].

The diagnosis of Paget’s disease relies heavily on histopathological and immunohistochemical analysis. MPD is distinguished by Paget cells within the nipple epidermis, frequently confirmed by markers such as cytokeratin 7 (CK7) and mucin 1 (MUC1), reflecting its breast cancer association [6]. EMPD, however, often expresses CK7 and mucin 5AC (MUC5AC), the latter distinguishing it from MPD and highlighting its apocrine gland origin [7]. Differential diagnosis is critical, as MPD must be differentiated from eczematous dermatitis or melanoma, while EMPD requires distinction from squamous cell carcinoma or Bowen’s disease, particularly in perianal or genital presentations [8]. Treatment evaluation has evolved significantly: MPD management now includes breast-conserving surgery and targeted therapies like HER2 inhibitors when applicable, whereas EMPD demands a tailored approach based on invasion depth and malignancy association, often involving surgical excision and long-term surveillance [9,10]. The additional dimension of EMPD lies in its variable prognosis, dependent on anatomical subtype and underlying malignancy, necessitating multidisciplinary care to optimize outcomes in these rare yet impactful conditions.

## Review

Epidemiology

MPD and EMPD are rare dermatological manifestations of adenocarcinoma, with comprehensive global epidemiological data remaining limited. The scarcity of large-scale studies underscores the reliance on regional investigations to estimate their prevalence, demographic patterns, and associated risk factors, highlighting the need for broader research to elucidate their worldwide distribution.

Prevalence

Specific prevalence data are sparse, but a population-based study in urban China in 2016 provided valuable insights. For MPD, the prevalence was reported at 0.42 per 100,000 population [11], approximately ten times higher than that of EMPD, which stood at 0.04 per 100,000 population in the same region and year [12]. These figures emphasize the rarity of both conditions, though MPD appears more common within this population. The introduction notes MPD’s association with underlying breast cancer and EMPD’s less frequent link to internal malignancies [3], but it lacks specific prevalence rates, making the Chinese data a critical addition.

Demographic Patterns

Demographic characteristics reveal distinct profiles for MPD and EMPD. The prevalence of MPD is reported as less than 5% of all breast cancers [11]. In a Chinese study, MPD exhibited a pronounced female predominance, with prevalence peaking among women aged 40-59 years and men aged 80 years and older [11]. Regional variations were notable, with the highest prevalence in Northwest China (1.21 per 100,000) and the lowest in Northeast China (0.06 per 100,000) [11]. Conversely, EMPD showed a slight male predominance, with prevalence rates of 0.05 per 100,000 in males versus 0.03 per 100,000 in females [12]. Its highest rate was observed in Southwest China (0.08 per 100,000), with a mean patient age of approximately 66 years and peak prevalence in the 70-79 age group [12]. These patterns suggest age and sex-specific differences that may reflect underlying etiological or hormonal influences (Table 1).

**Table 1:** Key demographic and epidemiologic features of mammary versus extramammary Paget's disease. Table 1 outlines the differences and similarities between MPD and EMPD. MPD has a prevalence of 0.42 per 100,000 (urban China, 2016), primarily affects women aged 40-59 and men over 80, varies regionally (highest in Northwest China at 1.21 per 100,000), and is strongly associated (>90%) with underlying breast carcinoma. In contrast, EMPD is rarer (0.04 per 100,000), slightly more common in men (0.05 vs. 0.03 per 100,000), peaks in older adults (70-79), shows higher prevalence in Southwest China (0.08 per 100,000), and affects apocrine-rich areas (e.g., perianal, genital, axillary), with variable links to malignancies like colorectal adenocarcinoma.

Feature	Mammary Paget's disease	Extramammary Paget's disease
Prevalence	0.42 per 100,000 (urban China, 2016) [11].	0.04 per 100,000 (urban China, 2016) [12].
Gender distribution	Marked female predominance [11].	Slight male predominance (0.05 vs. 0.03 per 100,000) [12].
Age distribution	Peaks in females aged 40–59 years, males aged 80+ [11].	Mean age ~66 years, peak in 70–79 age group [12].
Regional variation	Highest in Northwest China (1.21 per 100,000), lowest in Northeast China (0.06 per 100,000) [11].	Highest in Southwest China (0.08 per 100,000) [12].
Anatomic locations	Nipple-areolar complex [1].	Apocrine-rich areas: perianal, genital (e.g., vulvar, penoscrotal), axillary, etc. [2,10].
Association with underlying malignancies	Almost invariably associated with underlying breast carcinoma (>90% of cases) [1].	Variable; subtype-dependent (e.g., perianal EMPD with colorectal adenocarcinoma) [3,10].

Risk Factors

Both genetic and hormonal risk factors have been identified for MPD and EMPD, with some overlap and distinct features. For MPD, frequent mutations in chromatin remodeling genes, such as lysine methyltransferase 2C (KMT2C) and AT-rich interaction domain 2 (ARID2), are well-documented [4], aligning with its strong association with underlying breast carcinoma. Hormonal influences are also significant, as MPD frequently expresses estrogen receptors (ER) [13]. In EMPD, genetic alterations include mutations in the phosphoinositide 3-kinase-AKT-mammalian target of rapamycin (PI3K-AKT-mTOR) signaling pathway [14] and mismatch repair genes, with evidence of microsatellite instability [15]. Additionally, dysregulation of forkhead box A1 (FOXA1), observed in both MPD and EMPD, points to a hormonal component in their pathogenesis [16]. Environmental factors, such as chronic irritation in apocrine-rich areas for EMPD, are hypothesized but remain less substantiated. These risk factors, while partially overlapping with molecular insights from the introduction, provide a specific epidemiological perspective by linking genetic and hormonal elements to disease occurrence [4,5].

In summary, MPD and EMPD are exceedingly rare, with prevalence and demographic data primarily derived from regional studies like those in urban China. Their distinct age, sex, and regional patterns, alongside genetic and hormonal risk factors, underscore their complex epidemiology. Further global research is essential to refine these estimates and enhance our understanding of these uncommon conditions.

Pathogenesis and pathophysiology

**Figure 2:**
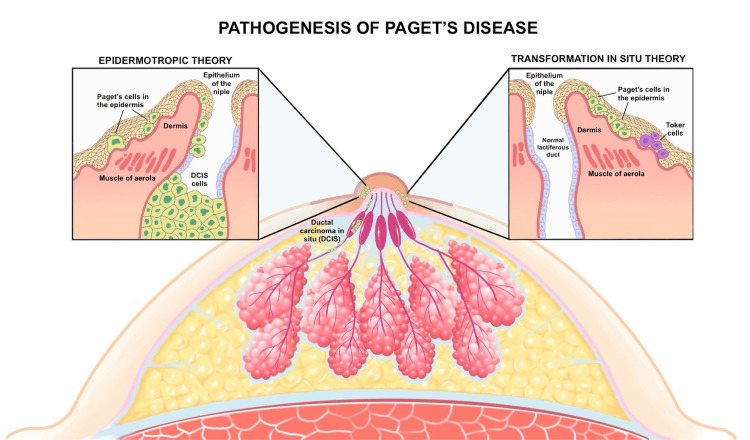
Pathogenesis Theories of Paget’s Disease This figure illustrates the two primary theories of Paget’s disease pathogenesis: the epidermotropic theory and the transformation in situ theory. The left side depicts the epidermotropic theory, where Paget cells migrate from an underlying DCIS within the lactiferous ducts, through the areola’s muscle and dermis, to the nipple epidermis. It shows Paget cells in the epidermis alongside normal ductal cells in the ducts. The right side represents the transformation in situ theory, where Paget cells originate directly within the nipple epidermis, independent of underlying DCIS, featuring Paget cells and Toker cells in the epithelium with a normal lactiferous duct. Image credit: Katherine López Soto.

The pathogenesis of MPD and EMPD revolves around the origin of Paget cells, with two prevailing theories: in situ transformation and epidermotropic migration. For MPD, the epidermotropic migration theory predominates, proposing that Paget cells are ductal carcinoma cells that travel from an underlying breast carcinoma- typically, ductal carcinoma in situ (DCIS) or invasive ductal carcinoma (IDC) to the nipple-areola complex epidermis [17,18]. This is bolstered by the consistent detection of breast cancer markers in Paget cells and the observation that over 90% of MPD cases are associated with such malignancies [19]. Conversely, the in situ transformation theory posits that Paget cells may originate independently within the epidermis, a hypothesis considered in rare instances where no underlying carcinoma is identified or when it is anatomically distant from the nipple (Figure 2) [17,20].

In EMPD, which affects apocrine-rich areas like the vulva, perianal region, and axilla, the origin of Paget cells is more heterogeneous. Evidence suggests an extraepidermal source, potentially from underlying adnexal structures such as apocrine or eccrine sweat glands or anogenital mammary-like glands [21,22]. However, the in situ transformation theory gains traction in some EMPD cases, supported by studies implicating the Msi1-mTOR pathway in converting keratinocytes into Paget-like cells [5]. Genetic analyses further complicate the picture, revealing that intraepidermal Paget cells can be genetically distinct from underlying carcinomas, indicating that pathogenesis may not always align with the epidermotropic model [18]. Thus, the development of Paget’s disease likely reflects a blend of these mechanisms, modulated by the presence and nature of associated malignancies.

The pathophysiology of MPD and EMPD is driven by distinct molecular pathways and markers. HER2 is a key player in MPD, with frequent overexpression and gene amplification observed, correlating with aggressive disease behavior and guiding the use of targeted therapies like trastuzumab [23,24]. In EMPD, HER2 overexpression is less common but significant in invasive cases, where it is linked to adverse prognostic factors such as lymph node metastasis [24]. Hormonal receptors also contribute: while ER expression is limited in both MPD and EMPD, androgen receptor (AR) expression is more frequent and associated with improved survival in MPD, hinting at potential hormonal therapy applications [23,25]. The PI3K-AKT-mTOR signaling pathway, marked by frequent activating mutations in EMPD, enhances cell proliferation and survival, distinguishing its pathophysiology from MPD [14,26]. The Msi1-mTOR pathway, active in both conditions, further bridges pathogenesis and pathophysiology by promoting Paget cell transformation, with preclinical mTOR inhibition showing therapeutic promise [5]. These molecular features, beyond those already noted in the epidemiology section (e.g., chromatin remodeling gene mutations), underscore the complexity of Paget’s disease and highlight actionable targets for intervention [4].

The strong association of MPD with underlying malignancies, particularly DCIS or IDC, shapes its clinical implications. Over 90% of MPD cases coexist with these breast cancers, often reflecting more aggressive tumor traits and worse survival outcomes compared to breast cancer alone [19,27]. This link necessitates comprehensive diagnostic approaches, including magnetic resonance imaging (MRI) to assess disease extent and tailored treatments addressing the underlying malignancy [28]. In EMPD, the connection to underlying cancers is less uniform but notable, especially in perianal or genital cases tied to adnexal or internal malignancies [21]. This variability emphasizes the need for individualized evaluation and management strategies, distinct from the diagnostic and treatment frameworks outlined earlier in the article, to address the specific pathophysiological context of each case.

Clinical presentation

MPD and EMPD exhibit distinct clinical features influenced by their anatomical locations and associations with underlying malignancies. MPD typically manifests as chronic eczematous changes in the nipple-areola complex, including redness, scaling, itching, and occasionally ulceration or bleeding [17,28]. These symptoms often persist for months to years, frequently mimicking benign dermatoses, which can delay diagnosis [17]. Up to 80-90% of MPD cases are linked to underlying DCIS or IDC, underscoring the need for prompt investigation when symptoms are unresponsive to topical treatments [17,28,29]. In contrast, EMPD presents as a slowly enlarging, erythematous, well-demarcated plaque in apocrine-rich regions such as the vulva, perianal area, and axilla, often accompanied by pruritus or pain [30,31]. These lesions also resemble inflammatory conditions, contributing to diagnostic delays that may span years [30,32].

The presentation varies significantly by location. In the nipple, MPD’s eczematous changes are a hallmark, strongly associated with underlying breast malignancies [17,28]. Vulvar EMPD, frequently asymptomatic, has a mean diagnostic delay of 25.1 months, with most cases remaining intraepidermal, though recurrences are common and typically confined to skin and mucosa [10]. Perianal EMPD, while similar in appearance, demonstrates a higher rate of invasive disease (50%) and a notable recurrence rate, with one-third of recurrences involving regional or distant metastases [10,33]. The chronicity of both MPD and EMPD lesions, often enduring for extended periods before diagnosis, highlights the importance of a high clinical suspicion and comprehensive evaluation to distinguish them from benign conditions and assess for associated malignancies [17,30,32].

Diagnosis and differential diagnosis

Diagnosing MPD and EMPD requires a combination of clinical assessment, imaging, and histopathological confirmation to identify Paget cells and rule out mimicking conditions. For MPD, clinical findings center on eczematous nipple changes, while imaging modalities enhance the detection of underlying malignancies. Mammography may reveal skin thickening, nipple retraction, subareolar or diffuse malignant microcalcifications, or discrete masses, though it is negative in up to 50% of cases [19,34]. Ultrasound complements this by identifying lobulated or irregularly contoured masses without posterior acoustic shadowing, often with increased blood flow in the affected nipple on Doppler, aiding differentiation from benign lesions [19,35]. MRI is particularly effective in detecting abnormal nipple enhancement, thickening of the nipple-areola complex, and associated DCIS or IDC, even when mammography and ultrasound are inconclusive [19,36,37].

For EMPD, diagnostic techniques focus on assessing disease extent and detecting associated malignancies. Pelvic MRI excels in delineating soft tissue involvement, showing well-enhanced lesions on gadolinium-enhanced T1-weighted imaging and hyperintense areas on diffusion-weighted imaging, which correlate closely with pathological findings, making it ideal for preoperative planning [38]. Computed tomography (CT), especially positron emission tomography (PET)/CT, is valuable for identifying nodal and distant metastases, though its effectiveness depends on tumor thickness and metastatic fluorodeoxyglucose (FDG) avidity [39]. Multiple biopsies, including mapping biopsies, are critical for histopathological confirmation and margin delineation, particularly in cases with ill-defined borders, and have been shown to reduce local recurrence rates when employed preoperatively [40,41].

Histopathologically, both MPD and EMPD feature large, atypical Paget cells with abundant pale cytoplasm and prominent nuclei within the epidermis, distinguishing them from the inflammatory profiles of psoriasis and eczema, which lack such cells [6]. Immunohistochemistry further refines the diagnosis: CK7 is consistently positive in Paget cells, contrasting with melanoma in situ, which is CK7-negative and positive for S100 and human melanoma black-45 (HMB-45) [6,42]. MPD typically expresses MUC1, while EMPD often expresses MUC5AC, providing a key differential marker between the two [7,43]. Additionally, p63 staining, typically negative in Paget’s disease but positive in pagetoid squamous cell carcinoma in situ, aids in distinguishing these conditions [44]. This combination of histopathological and immunohistochemical features ensures accurate diagnosis and differentiation from other dermatological entities.

Management and treatment

Managing MPD and EMPD demands a customized approach, given that each presents its own clinical nuances and varying degrees of association with underlying cancers.

Surgical Treatment Options for Mammary Paget’s Disease

For MPD, surgical intervention remains the cornerstone of treatment, with the choice between mastectomy and breast-conserving surgery (BCS) with radiotherapy determined by disease extent, the presence of DCIS or IDC, and patient preferences. Mastectomy, historically the standard approach, involves complete breast removal and is favored in cases with extensive underlying cancer or when clear margins are unattainable with BCS [1]. It ensures comprehensive tumor clearance, particularly in advanced presentations. Conversely, BCS with radiotherapy has emerged as a feasible alternative for select patients, involving excision of the nipple-areolar complex and any associated malignancy, followed by radiotherapy to minimize local recurrence. Studies demonstrate that BCS with radiotherapy yields oncological outcomes comparable to mastectomy in MPD cases linked to DCIS, with careful margin assessment being paramount [45,46,47]. In patients with IDC, BCS remains viable but requires meticulous selection due to elevated recurrence risks [45,48]. This approach preserves breast aesthetics and function, offering a safe option when negative margins are achieved and adjuvant therapies are appropriately administered [45,46,49]. The decision between these options should be individualized, balancing oncologic efficacy with patient-specific goals.

Systemic Therapy for Mammary Paget’s Disease

Systemic therapy, including chemotherapy, hormonal therapy, and anti-HER2 therapy, is reserved for MPD cases associated with invasive or advanced breast cancer, guided by tumor stage and molecular profile. Chemotherapy is indicated for high-grade invasive disease, lymph node involvement, or metastatic spread, aiming to control systemic progression [50]. Hormonal therapy, such as tamoxifen or aromatase inhibitors, is employed in hormone receptor-positive cases, tailored to menopausal status to target estrogen-driven growth [50]. Anti-HER2 therapy, notably trastuzumab, is critical for HER2-positive MPD, where overexpression or amplification, confirmed via immunohistochemistry or in situ hybridization, dictates its use per American Society of Clinical Oncology (ASCO) guidelines [50,51]. These therapies address the underlying malignancy’s biological drivers, enhancing outcomes beyond surgical resection. Treatment selection hinges on receptor status, disease extent, and patient comorbidities, ensuring a personalized approach that complements local management strategies.

Treatment Approaches for Extramammary Paget’s Disease

EMPD, affecting apocrine-rich regions like the vulva, perianal area, and axilla, demands a multifaceted treatment approach based on disease localization and invasiveness. Wide local excision (WLE) is the primary modality for resectable EMPD, targeting complete tumor removal with clear margins. However, the multifocal nature of EMPD often complicates margin clearance, making Mohs micrographic surgery (MMS) a preferred alternative due to its superior margin control and reduced recurrence rates (Table 2) [52,53,54].

**Table 2:** Comparison of surgical approaches in mammary Paget’s disease and extramammary paget’s disease. iven here is the comparison of surgical approaches for MPD and EMPD, outlining techniques, descriptions, outcomes and reconstruction methods. For MPD, mastectomy entails complete breast removal, including the nipple-areolar complex, providing high local control but with significant cosmetic and functional impact, with reconstruction options like implants or autologous tissue (e.g., DIEP flap). Alternatively, BCS with radiotherapy removes the nipple-areolar complex and underlying cancer, followed by radiotherapy, offering comparable outcomes to mastectomy in selected cases (especially with DCIS) while preserving breast tissue, using oncoplastic techniques and nipple reconstruction. For EMPD, WLE excises the tumor with margins, with outcomes varying based on margin status and reconstruction via primary closure, skin grafts, or local flaps (e.g., pudendal thigh flap for vulvar EMPD). In contrast, MMS employs layer-by-layer excision with immediate margin assessment, achieving better margin control and lower recurrence rates than WLE, with similar reconstruction options. This summary highlights the distinct surgical strategies tailored to each disease, balancing oncologic efficacy with aesthetic and functional outcomes. MPD: Mammary Paget’s disease; EMPD: extramammary Paget’s disease; BCS: breast-conserving surgery; WLE: wide local excision; MMS: Mohs micrographic surgery; DIEP: deep inferior epigastric perforator.

Disease	Surgical technique	Description	Outcomes	Reconstruction techniques	References
MPD	Mastectomy	Removal of the entire breast, including the nipple-areolar complex.	High local control rates; significant cosmetic and functional impact.	Implant-based reconstruction, autologous tissue reconstruction (e.g., DIEP flap).	[45,48]
MPD	BCS with radiotherapy	Excision of the nipple-areolar complex and underlying cancer, followed by radiotherapy.	Comparable outcomes to mastectomy in selected cases, especially with DCIS.	Oncoplastic techniques, nipple reconstruction.	[45,46,47,49]
EMPD	WLE	Surgical removal of the tumor with a margin of normal tissue.	Variable outcomes; recurrence rates depend on margin status.	Primary closure, skin grafts, local flaps (e.g., pudendal thigh flap for vulvar EMPD).	[52,54]
EMPD	MMS	Layer-by-layer excision with immediate microscopic margin assessment.	Better margin control, lower recurrence rates compared to WLE.	Similar to WLE: primary closure, skin grafts, local flaps.	[52,53,54]

Radiotherapy serves as an option for non-surgical candidates or extensive disease, functioning as definitive treatment, adjuvant therapy, or palliation for recurrence, with doses ≥60 Gy optimizing local control [55,56]. Topical treatments, such as imiquimod or 5-fluorouracil, offer non-invasive alternatives for superficial EMPD or patients unfit for surgery, with imiquimod achieving a complete response in approximately 54% of cases [57]. Less effective options like photodynamic therapy (PDT) and laser treatments are considered in specific contexts but lack robust efficacy data [57]. Treatment decisions should reflect disease characteristics, patient health, and potential underlying malignancies, often necessitating a multidisciplinary framework to balance efficacy and morbidity.

Long-Term Follow-Up and Surveillance

Post-treatment surveillance for MPD and EMPD is essential to monitor for recurrence and secondary malignancies, tailored to each condition’s natural history. For MPD, follow-up aligns with breast cancer protocols, typically involving clinical examinations and imaging (e.g., mammography or MRI) every 6-12 months for the first few years, then annually, to detect local recurrence or new primaries early [28]. In EMPD, the chronicity and recurrence risk, particularly with positive margins, demand sustained vigilance through regular dermatological assessments and imaging for suspected invasion or metastasis [52]. Sentinel lymph node biopsy (SLNB) may guide management in invasive EMPD by identifying subclinical nodal spread [58]. Additionally, the heightened risk of secondary malignancies, notably colorectal or anal cancers in invasive EMPD, warrants prolonged screening [59]. Surveillance strategies should be customized to initial treatment outcomes, disease invasiveness, and patient factors, supported by multidisciplinary collaboration to optimize long-term outcomes.

Prognosis and outcomes

When it comes to the outlook for MPD and EMPD, several unique factors shape how the disease advances, its chances of coming back, and overall survival.

Prognostic Factors

Several prognostic factors shape outcomes in MPD and EMPD, encompassing disease extent, histological characteristics, and molecular markers. In MPD, the extent of disease, reflected by pathological T (tumor size) and N (lymph node) stages, is a primary determinant of prognosis, with advanced stages correlating with increased recurrence and mortality risk. AR positivity, observed in a subset of MPD cases, is associated with improved overall survival (OS), suggesting a protective role that may guide future therapeutic strategies [25]. HER2 overexpression, present in approximately 78.2% of MPD cases, is another critical factor, often linked to aggressive behavior and responsiveness to targeted therapies, thus influencing both prognosis and treatment planning [25].

For EMPD, lymphovascular invasion and surgical margin status are pivotal prognostic indicators. Lymphovascular invasion signals a heightened risk of regional and distant spread, while positive surgical margins correlate with increased local recurrence. HER2 overexpression, though less frequent than in MPD, is significant in EMPD, particularly in invasive cases, where it is associated with deeper tumor invasion and lymph node metastasis, marking a more aggressive disease course [24,60]. Lymph node status further refines prognosis in EMPD, serving as a robust predictor of survival [24]. These factors collectively underscore the importance of thorough histopathological assessment and molecular profiling to tailor management and predict outcomes effectively.

Local Recurrence and Metastasis Rates

Local recurrence and metastasis rates differ substantially between MPD and EMPD, reflecting their unique biological profiles and treatment responses. In MPD, local recurrence varies by surgical approach: BCS alone yields a recurrence rate of 21.2%, whereas mastectomy and BCS with radiotherapy reduce this to 5.9% and 8%, respectively [48]. The presence of underlying invasive carcinoma amplifies metastatic risk, with palpable tumors linked to a 30.2% metastasis rate compared to 3.4% for impalpable tumors, highlighting the prognostic weight of associated malignancy [48].

EMPD exhibits higher local recurrence rates overall, particularly with WLE. Metastasis in EMPD, while less frequent, occurs in 17% of cases, with invasive disease and positive lymph nodes as key predictors of systemic spread [52,61]. These disparities emphasize the need for optimized surgical techniques and vigilant post-treatment surveillance, tailored to each condition’s recurrence and metastatic potential.

Impact of Underlying Carcinoma on Survival and Quality of Life

The presence of an underlying carcinoma profoundly influences survival and quality of life, particularly in MPD, where over 90% of cases are associated with DCIS or IDC [19]. Patients with MPD and underlying invasive breast carcinoma (IBC) experience significantly worse survival compared to those with IBC alone, driven by higher lymph node involvement, reduced hormone receptor expression, and elevated HER2 positivity. A matched cohort study reported a 5-year relapse-free survival (RFS) of 52.2% and OS of 62.1% for MPD with IBC, versus 81.4% and 85.9% for IBC controls, underscoring the adverse prognostic impact [62]. This poorer outlook often necessitates aggressive treatments such as mastectomy, chemotherapy, or anti-HER2 therapy, which introduce physical morbidities, including loss of breast function and treatment-related toxicities, alongside a psychological burden from the graver prognosis, collectively diminishing quality of life [27,62,63].

In EMPD, the association with underlying malignancy is less consistent but impactful when present, particularly in perianal or genital cases linked to adnexal or internal cancers. While specific survival data for EMPD with underlying carcinoma are less delineated in the provided text, the increased metastatic potential and recurrence risk in invasive cases suggest a parallel decline in survival and quality of life, compounded by extensive surgical and systemic interventions [52,61]. These findings highlight the need for comprehensive supportive care to mitigate the multifaceted challenges posed by underlying carcinoma in both MPD and EMPD.

Recent advances and research directions

Recent advances in our understanding of MPD and EMPD have radically shifted how we approach these conditions. Breakthroughs in diagnostic techniques and targeted therapies fueled by deeper molecular and genetic insights now allow clinicians to detect these diseases earlier and design treatment plans that are finely tuned to each patient’s specific profile.

New Diagnostic Technologies and Molecular Studies

Emerging diagnostic technologies, particularly those rooted in molecular analyses, are transforming the detection and characterization of Paget’s disease. Whole-Exome Sequencing (WES) has identified recurrent mutations in chromatin remodeling genes, such as KMT2C and ARID2, in both MPD and EMPD, suggesting these alterations are early drivers of disease development [4]. These mutations not only deepen our understanding of pathogenesis but also hold potential as diagnostic biomarkers and therapeutic targets. Complementing this, single-cell RNA sequencing (scRNA-seq) has illuminated the role of the Msi1-mTOR pathway, revealing distinct cellular states and novel biomarkers that point to mTOR inhibition as a viable treatment strategy [5]. Additionally, immunohistochemical techniques have advanced the classification of MPD, uncovering a predominance of the HER2-enriched subtype, which facilitates the selection of targeted therapies like HER2 inhibitors [64]. These molecular tools enhance diagnostic precision beyond traditional histopathology, offering insights into disease biology that were previously inaccessible and paving the way for more effective interventions.

Clinical Trials on Immunotherapy and Novel Targeted Treatments

Clinical research into immunotherapy and targeted therapies is gaining momentum, particularly for EMPD, driven by molecular profiling that identifies actionable treatment avenues. Studies suggest that EMPD patients with low Erb-B2 receptor tyrosine kinase 2 (ERBB2) (HER2) expression exhibit heightened immunogenicity and enriched immune pathways, making them prime candidates for B-cell-related immunotherapy [65]. This finding highlights immunotherapy’s potential as a novel approach, especially in HER2-low cases where conventional targeted therapies may falter. Although the rarity of Paget’s disease limits large-scale trials, these investigations emphasize the importance of tailoring treatments to molecular characteristics, such as HER2 status and immune pathway activation. Ongoing efforts aim to validate these strategies, potentially expanding the therapeutic arsenal for both MPD and EMPD.

Precision Medicine and Molecular Subgroup Identification

Precision medicine is reshaping the management of Paget’s disease by identifying molecular subgroups that guide therapy selection. In EMPD, low ERBB2 expression correlates with immunological features that suggest greater responsiveness to immunotherapy, enabling stratified treatment approaches [65]. These advances underscore the value of molecular profiling in distinguishing patient cohorts likely to benefit from specific therapies, moving beyond generic treatment paradigms. By aligning interventions with disease biology such as HER2 status or immune pathway enrichment, precision medicine enhances therapeutic efficacy and offers a personalized framework for managing MPD and EMPD. Continued research seeks to refine these molecular classifications and broaden their clinical application.

## Conclusions

This review navigates the complexity of MPD and EMPD, outlining their evolving definitions and exploring various diagnostic and therapeutic approaches. From advanced tools like molecular profiling and imaging to surgical interventions such as mastectomy for MPD and MMS for EMPD, each strategy presents distinct advantages and limitations. While not exhaustive, this concise review captures the essence of the most relevant aspects in the evolving landscape of these rare diseases, providing valuable insights for clinicians. To determine the optimal strategies, further investigations are crucial. Comparative studies assessing the efficacy, recurrence rates, and patient outcomes of each approach, along with considerations for specific disease characteristics and comorbidities, are necessary. A comprehensive understanding of the evolving landscape of MPD and EMPD will guide clinicians in choosing the most effective and personalized approach for individual cases.
